# Division of the role and physiological impact of multiple lysophosphatidic acid acyltransferase paralogs

**DOI:** 10.1186/s12866-022-02641-8

**Published:** 2022-10-06

**Authors:** Takuya Ogawa, Misaki Kuboshima, Nittikarn Suwanawat, Jun Kawamoto, Tatsuo Kurihara

**Affiliations:** Institute for Chemical Research, Kyoto University, Gokasho, Uji, Kyoto, 611-0011 Japan

**Keywords:** Lysophosphatidic acid acyltransferase, PlsC, YihG, Phospholipid, Fatty acid composition

## Abstract

**Background:**

Lysophosphatidic acid acyltransferase (LPAAT) is a phospholipid biosynthesis enzyme that introduces a particular set of fatty acids at the *sn*-2 position of phospholipids. Many bacteria have multiple LPAAT paralogs, and these enzymes are considered to have different fatty acid selectivities and to produce diverse phospholipids with distinct fatty acid compositions. This feature is advantageous for controlling the physicochemical properties of lipid membranes to maintain membrane integrity in response to the environment. However, it remains unclear how LPAAT paralogs are functionally differentiated and biologically significant.

**Results:**

To better understand the division of roles of the LPAAT paralogs, we analyzed the functions of two LPAAT paralogs, PlsC4 and PlsC5, from the psychrotrophic bacterium *Shewanella livingstonensis* Ac10. As for their enzymatic function, lipid analysis of *plsC4*- and *plsC5*-inactivated mutants revealed that PlsC4 prefers *iso*-tridecanoic acid (C_12_-chain length, methyl-branched), whereas PlsC5 prefers palmitoleic acid (C_16_-chain length, monounsaturated). Regarding the physiological role, we found that *plsC4*, not *plsC5*, contributes to tolerance to cold stress. Using bioinformatics analysis, we demonstrated that orthologs of PlsC4/PlsC5 and their close relatives, constituting a new clade of LPAATs, are present in many γ-proteobacteria. We also found that LPAATs of this clade are phylogenetically distant from principal LPAATs, such as PlsC1 of *S. livingstonensis* Ac10, which are universally conserved among bacteria, suggesting the presence of functionally differentiated LPAATs in these bacteria.

**Conclusions:**

PlsC4 and PlsC5, which are LPAAT paralogs of *S. livingstonensis* Ac10, play different roles in phospholipid production and bacterial physiology. An enzyme belonging to PlsC4/PlsC5 subfamilies and their close relatives are present, in addition to principal LPAATs, in many γ-proteobacteria, suggesting that the division of roles is more common than previously thought. Thus, both principal LPAATs and PlsC4/PlsC5-related enzymes should be considered to decipher the metabolism and physiology of bacterial cell membranes.

**Supplementary Information:**

The online version contains supplementary material available at 10.1186/s12866-022-02641-8.

## Background

**Fig. 1:**
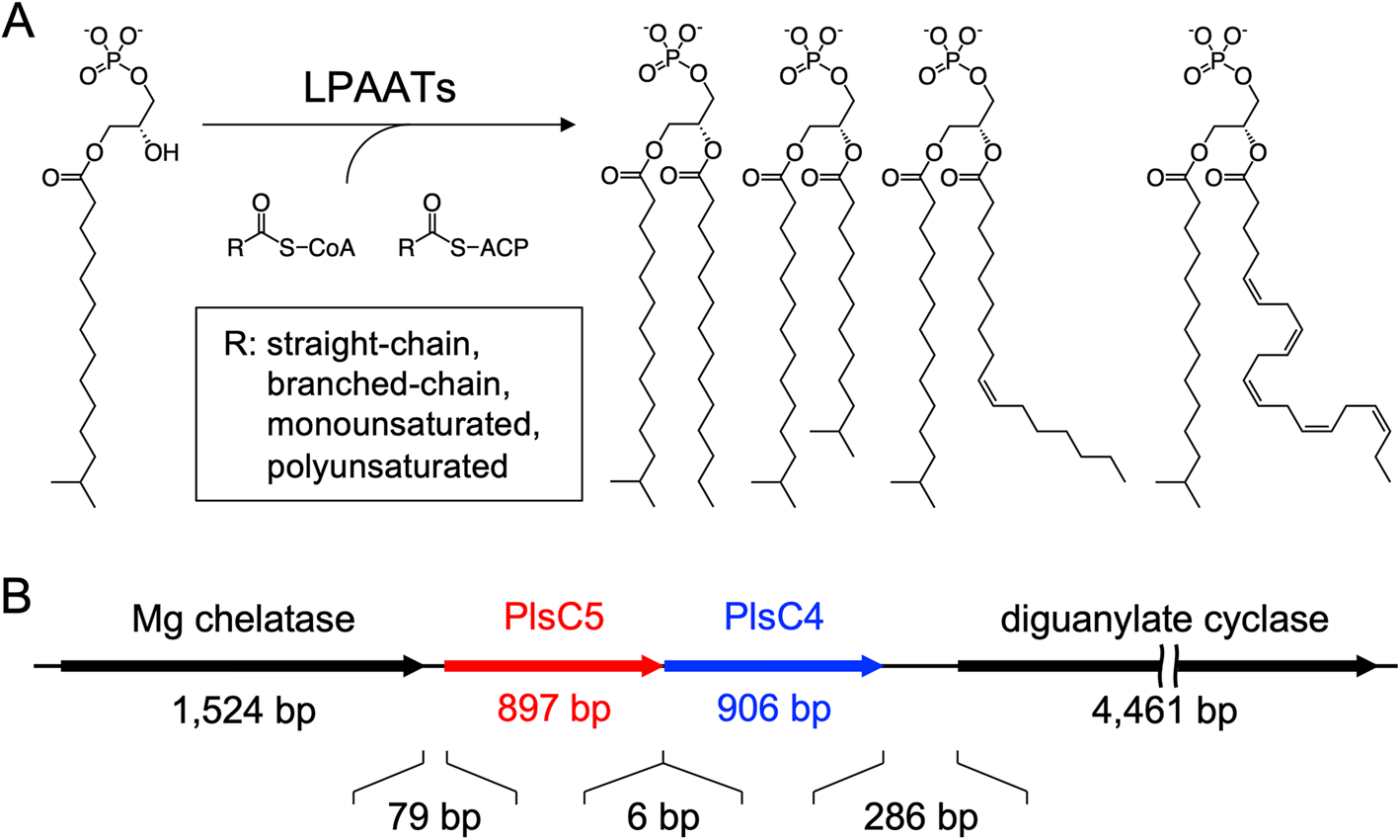
Schematic illustration of the LPAAT reaction (**A**) and genomic organization of *plsC4*, *plsC5*, and flanking genes (**B**). R, hydrocarbon chain; CoA, coenzyme A; ACP, acyl carrier protein

In bacteria, fatty acyl groups are introduced into phospholipids (PLs) by the sequential action of two enzymes*, sn*-glycerol-3-phosphate acyltransferase and lysophosphatidic acid acyltransferase (LPAAT) [1]. The latter enzyme acylates the *sn*-2 position of lysophosphatidic acid (LPA) to yield phosphatidic acid (PA) (Fig. 1A) [2]. PA is subsequently subjected to modification of the *sn*-3 polar head group, which generates mature membrane PLs such as phosphatidylethanolamine (PE) and phosphatidylglycerol (PG). The LPAAT-coding gene is typically designated as *plsC*. Importantly, more than one *plsC* gene is often present in a single bacterial species, and a few studies have reported that a mutant of each *plsC* produces PLs with different fatty acid compositions [3–6], suggesting that each PlsC paralog has unique substrate selectivity towards fatty acids. This leads to the production of distinct PLs, thereby diversifying the fatty acid composition of PL membranes. The carbon chain length, degree of unsaturation, and/or methyl branching of the fatty acyl groups of PLs significantly contribute to the physicochemical properties of the membrane (e.g., fluidity and permeability) [7, 8] and folding and function of transmembrane proteins that interact with PLs [9, 10]. Therefore, the presence of multiple PlsC paralogs may be physiologically relevant. Consistent with this hypothesis, mutations in some *plsC* genes alter not only the PL composition, but also affect cell physiology [5].

In addition, although *Escherichia coli* was considered to have one LPAAT encoded by *plsC* because its deletion is lethal [11], we recently demonstrated that *yihG* from *E. coli* codes for a second LPAAT and can substitute for *plsC* when it is overexpressed (therefore, we consider that it is reasonable to regard PlsC as a “principal” LPAAT, not the only LPAAT) [12]. YihG is a distant homolog of PlsC, with only 18% amino acid sequence identity, and shares several motifs of the PL acyltransferase. YihG exhibits substrate selectivity different from that of PlsC, and a *yihG*-deleted mutant is more motile than a wild-type cell. A BLAST search indicated that other bacteria, such as *Pseudomonas* and *Vibrio* also have YihG ortholog(s). Thus, YihG is emerging as a new member of the LPAAT family that diversifies the fatty acid composition of the bacterial cell membrane. However, it is not yet clear how individual LPAAT paralogs and their PL products play a role in cellular physiology.

The Antarctic marine bacterium *Shewanella livingstonensis* Ac10 possesses five LPAAT paralogs, which are designated as PlsC1–PlsC5 and exhibit PL acyltransferase motifs [13]. Among these paralogs, PlsC1 exhibits the highest homology to *E. coli* PlsC (45% identical), while the others show less homology (17–21%). PlsC4 and PlsC5 are more homologous to *E. coli* YihG (39 and 38%, respectively) than to *E. coli* PlsC (21 and 17%, respectively). Assuming that these paralogs play distinct roles in both PL formation and physiology, we have studied the biological importance of the presence of multiple LPAATs and their corresponding PL products. We characterized PlsC1 in vivo and in vitro and demonstrated that PlsC1 preferentially acylates LPA with eicosapentaenoic acid (20:5, EPA) [13, 14]. EPA-containing PLs are important for the cell division of *S. livingstonensis* Ac10 in cold environments [15] and have antioxidant activity and molecular chaperone-like functions [16, 17]. We also revealed the distinct function of PlsC4: when *plsC4* is disrupted, the amount of PLs that have 11-methyllauric acid (or *iso*-tridecanoic acid, i13:0) and 13-methylmyristic acid (or *iso*-pentadecanoic acid, i15:0) at the *sn*-2 position is reduced [18]. The physiological roles of branched-chain fatty acids (BCFAs)-containing PLs in *S. livingstonensis* Ac10 remain elusive. However, in the piezophilic bacterium *Shewanella piezotolerans* WP3, i13:0 and i15:0 levels are upregulated when grown at 4 °C, and BCFAs are required for proper growth at cold temperatures [19]. A BCFA-containing PL exhibits a lower phase transition temperature than the corresponding straight-chain PL and thus maintains the fluidity of the lipid membrane at cold temperatures [20].

The interesting characteristics of PlsC4 and PlsC5 are that the paralogs are 39% identical to each other and that the coding genes are tandemly aligned in the genome (Fig. 1B). The former feature would provide an insight into the mechanism of substrate selectivity through comparative analysis, while the latter suggests the co-production of these enzymes and their cooperative role in lipid metabolism or membrane-related physiology. We previously attempted to examine their functions by characterizing gene-disrupted mutants. A *plsC4*-disrupted mutant (Δ*plsC4*) exhibited decreased cell motility on a soft agar plate and abnormal cell flocculation in liquid culture (unpublished data). However, these phenotypes resulted from a polar effect on the downstream diguanylate cyclase gene (Fig. 1B), which regulates cell motility and biofilm formation [21–23]. Likewise, we also failed to determine the function of PlsC5 because of a polar effect on *plsC4* (Fig. 1B), and the *plsC5*-disrupted mutant had a Δ*plsC4*-like PL composition. To better understand the division of the role of LPAAT paralogs in bacteria, in this study we made *plsC4-* and *plsC5-*inactivated mutants (designated as *plsC4*^inact.^ and *plsC5*^inact.^ mutants, respectively) and characterized them. We confirmed that the mutants exhibited no appreciable polar effects and successfully determined the enzymatic functions of PlsC4 and PlsC5 in vivo along with the growth characteristics of these mutants. We also revealed that PlsC4/PlsC5 orthologs and their close relatives are present, in addition to principal LPAATs (e.g., PlsC from *E. coli* and PlsC1 from *S. livingstonensis* Ac10), in γ-proteobacteria, suggesting that the division of the role of multiple LPAAT paralogs is common and complex in various bacteria.

## Results

### Generation of *plsC4*^inact.^ and *plsC5*^inact.^ mutants

**Fig. 2:**
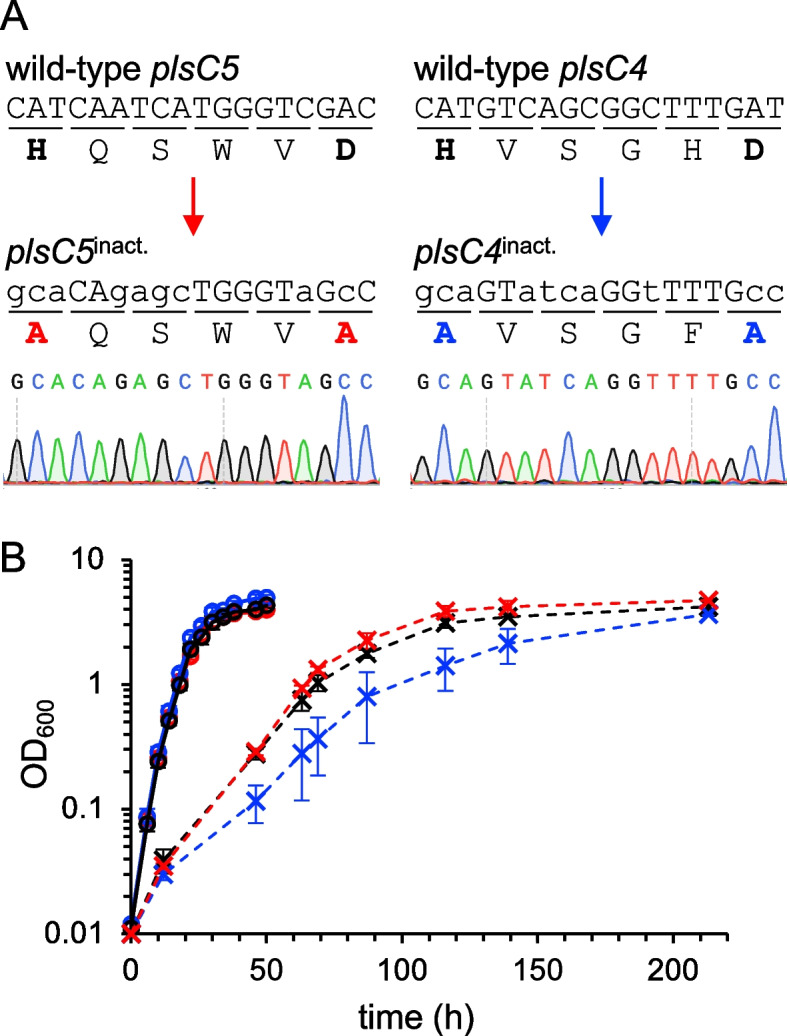
Construction of *plsC4*^inact.^ and *plsC5*^inact.^ mutants. **A** The wild-type (upper) and mutated (lower) nucleotide and amino acid sequences of catalytic site-coding regions. The small characters indicate the mutated nucleotides. A SalI site in the wild-type *plsC5*, of which loss indicates the successful mutation, is shaded. Raw sequencing data for the mutated sequences are shown at the bottom. **B** The growth curves of parent (black), *plsC4*^inact.^ (blue), and *plsC5*^inact.^ cells (red). Solid and broken lines indicate growth profiles at 18 °C and 4 °C, respectively

To analyze the enzymatic functions of PlsC4 and PlsC5 in vivo without unwanted polar effects, we inactivated PlsC4 and PlsC5 by introducing mutations to codons that code for the conserved acyltransferase motif I (i.e. His-Xaa-Xaa-Xaa-Xaa-Asp), where His and Asp residues are essential to catalysis [24, 25]. Silent mutations have also been introduced to facilitate the screening of mutants. Through a two-step single-crossover recombination method [26], we successfully introduced mutations to obtain *plsC4*^inact.^ and *plsC5*^inact.^ mutants expressing mutated PlsC4 and PlsC5, whose catalytic His and Asp residues were substituted with Ala (Fig. 2A).

The mutants were tested by a cell motility assay to examine whether the mutations induced a polar effect, as observed in the Δ*plsC4* mutant. Motility of *plsC4*^inact.^ cells, as well as *plsC5*^inact.^ cells, was comparable to that of the parent cells (Fig. S1). Abnormal cell flocculation, another consequence of the polar effect induced by *plsC4* disruption, was not observed with *plsC4*^inact.^ cells (data not shown). In addition, unlike the *plsC5*-disrupted mutant, with a PL composition similar to that of the Δ*plsC4* mutant which was attributed to the polar effect, *plsC5*^inact.^ cells displayed a unique PL pattern, as described below. Taken together, we confirmed that mutating only the catalytic site-coding sequence did not induce a polar effect.

Growth rate of *plsC4*^inact.^ cells was slightly slower than that of the parent and *plsC5*^inact.^ cells when cultured at 4 °C, whereas their growth was comparable at 18 °C (Fig. 2B). Given that PlsC4 produces BCFA-containing PLs, the growth retardation of *plsC4*^inact.^ cells at cold temperatures was consistent with the cold susceptibility of a Leu/Ile/Val uptake-deficient mutant of *S. piezotolerans*, which did not upregulate the BCFA levels at 4 °C [19].

### Electrospray ionization (ESI)-mass spectrometry (MS) analyses of PL compositions

The parent strain and *plsC4*^inact.^ and *plsC5*^inact.^ mutants that harbored either a control or complementation plasmid were cultured in LB media at 4 °C, and their PLs were extracted using the Bligh-Dyer method. The extracts were subjected to ESI-MS analysis, and the relative abundances of each PE and PG species were calculated from their ion intensities in the mass spectra. Although PAs are the direct products of the LPAAT reactions, the ion intensities of most PA molecules were not high enough to be quantified probably because most PAs were immediately converted into PEs and PGs. Therefore, we analyzed PE and PG profiles instead of a PA profile. Because PEs and PGs are produced from PAs via modification of the *sn*-3 head group and it is generally believed that acyl chain remodeling contributes little to the formation of PLs in bacteria, it is reasonable to consider that the acyl chain compositions of PEs and PGs reflect that of PAs. PLs are represented with the naming system where 32:1-PE, for instance, indicates a PE with 32 carbon atoms and one double bond in its acyl moiety. MS/MS analysis was performed to determine the fatty acid composition of the PL species. The positions of the fatty acyl groups were deduced based on the finding that the *sn*-2 fatty acyl group exhibits higher ion intensity than the *sn*-1 fatty acyl group in MS/MS spectra [27, 28].

**Fig. 3:**
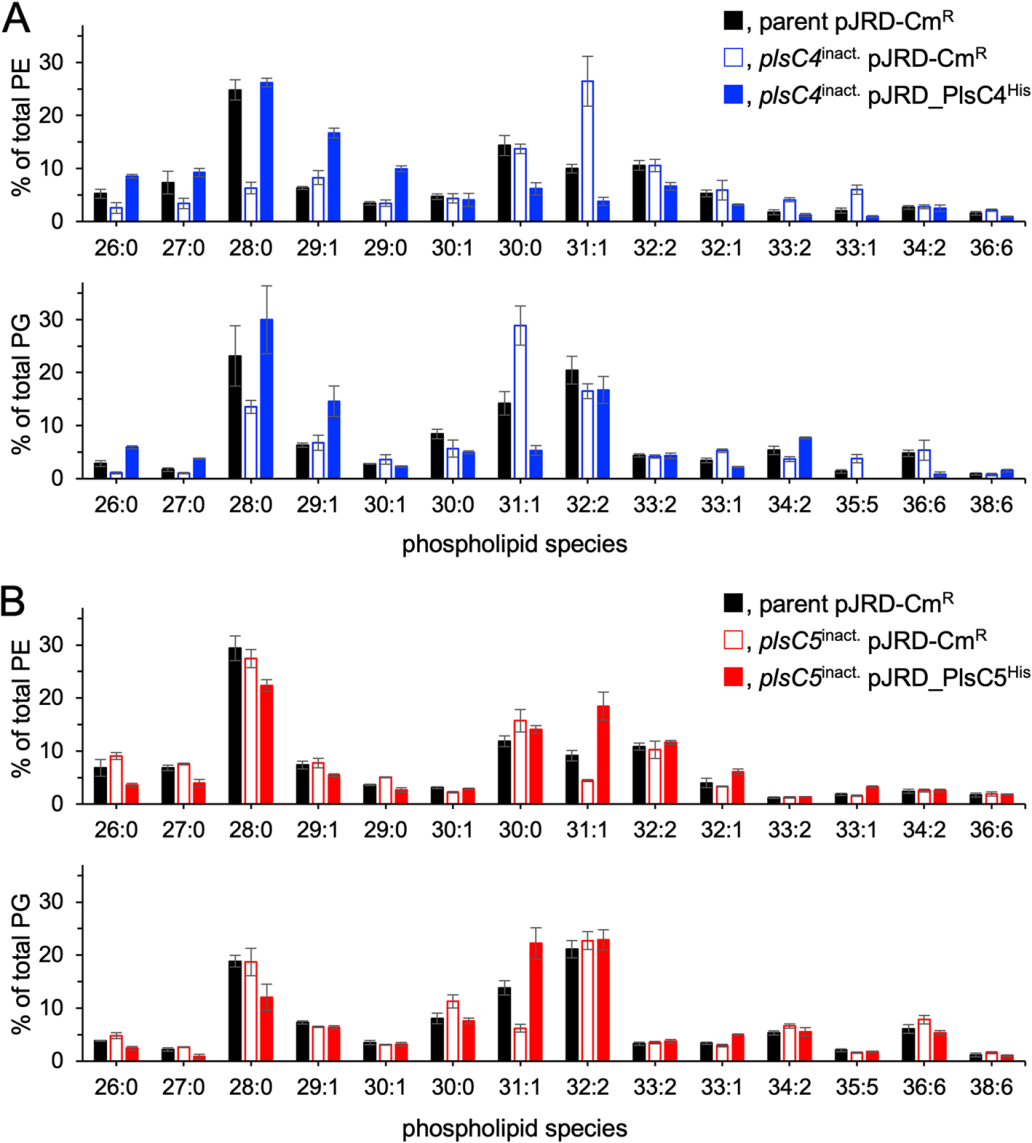
ESI-MS analysis of phospholipids. **A** Relative abundances of PE and PG species in the parent cells harboring pJRD-Cm^R^ (closed black bar) and *plsC4*^inact.^ cells harboring pJRD-Cm^R^ (open blue bar) and pJRD_PlsC4^His^ (closed blue bar). **B** Relative abundances of PE and PG species in the parent cells harboring pJRD-Cm^R^ (closed black bar) and *plsC5*^inact.^ cells harboring pJRD-Cm^R^ (open red bar) and pJRD_PlsC5^His^ (closed red bar). The data were obtained from three independent experiments

PL composition of the *plsC4*^inact.^ mutant clearly differed from that of the parent strain (Fig. 3A). Notably, PLs 28:0-PE and 28:0-PG were most abundant in the parent cells, accounting for 24.8 and 23.1% of total PEs and PGs, respectively, whereas they reduced to 6.3 and 13.5% in the *plsC4*^inact.^ mutant, respectively. The MS/MS analysis suggested that 28:0-PE (Fig. S2A) and 28:0-PG (data not shown) of both parent and mutant cells were acylated with i15:0 at the *sn*-1 position and i13:0 at the *sn*-2 position. PLs 26:0-PE, 27:0-PE, 26:0-PG, and 27:0-PG, which were composed of i13:0 at the *sn*-2 position and either i13:0 or myristic acid (14:0) at the *sn*-1 position (data not shown), were also markedly decreased in the mutant. These results indicate that PlsC4 incorporated i13:0 at the *sn*-2 position of the PLs. PE containing two i15:0 acyl groups (30:0-PE) (Fig. S2B), and the corresponding PG did not change as much as those in the Δ*plsC4* mutant, in which the amount of these PLs was reduced by approximately 70% [18]. In contrast, 31:1-PE and 31:1-PG, which are composed of i15:0 and palmitoleic acid (16:1) at the *sn*-1 and *sn*-2 positions, respectively (Fig. S3A, top), were increased by 2.0–2.7-fold and were most abundant in the *plsC4*^inact.^ mutant (Fig. 3A). The amount of several minor PLs, including 33:2-PE, 33:1-PE, 33:1-PG, and 35:5-PG, also increased.

These alterations were reversed when the complementation plasmid was introduced into the *plsC4*^inact.^ mutant (Fig. 3A). In addition, we noticed that 31:1-PE and 31:1-PG not only decreased, but also altered the fatty acid composition. Fig. S2C demonstrates that the 31:1-PE of the complemented cells produced an intense peak of i13:0 (and the same trend was observed for 31:1-PG (data not shown)), suggesting elevated levels of i13:0 at the *sn*-2 position compared with that in the parent cells. Likewise, 29:1-PE (Fig. S2D), as well as 29:0-PE and 29:1-PG (data not shown), which were overproduced in the complemented cells, primarily had i13:0 at the *sn*-2 position. These findings strongly suggest the preference of PlsC4 towards i13:0.

PL composition of the *plsC5*^inact.^ mutant was almost the same as that of the parent strain, except for 31:1-PE and 31:1-PG, which were approximately 50% lower in the mutant than that in the parent cells (Fig. 3B). Considering that the product ion of i15:0 was lower than that of 16:1 in the parental 31:1-PE (Fig. S3A, top) and 31:1-PG (data not shown), *sn*-2 was likely acylated with 16:1, whereas *sn*-1 with i15:0. In contrast, the ion intensities of i15:0 and 16:1 were comparable, and the additional product ions of i13:0 and vaccenic acid (18:1) appeared in the mutant lipids (Fig. S3A, middle). These findings suggested that 31:1-PE comprised a mixture of PEs containing i15:0 and 16:1, 16:1 and i15:0, and 18:1 and i13:0 at the *sn*-1 and *sn*-2 positions, respectively, in the *plsC5*^inact.^ cells. In addition, 29:1-PE and 32:1-PE had a reduced level of 16:1 at the *sn*-2 position in the mutant cells, despite their relative abundances in the parent and *plsC5*^inact.^ cells were comparable (Fig. S3B for the 29:1-PE). Complementation with *plsC5* led to the overproduction of 31:1-PE and 31:1-PG and the recovery of the 16:1 level at the *sn*-2 position (Fig. 3B, S3A, and S3B). Taken together, these results suggest that PlsC5 selectively incorporates 16:1 at the *sn*-2 position of PLs. However, it should be noted that the relative abundance of 32:2-PE, which had two 16:1 acyl groups (Fig. S3C), and the corresponding PG did not change upon inactivation or complementation (Fig. 3B).

### Gas chromatography (GC)-MS analysis of *sn*-2 fatty acyl groups

**Fig. 4:**
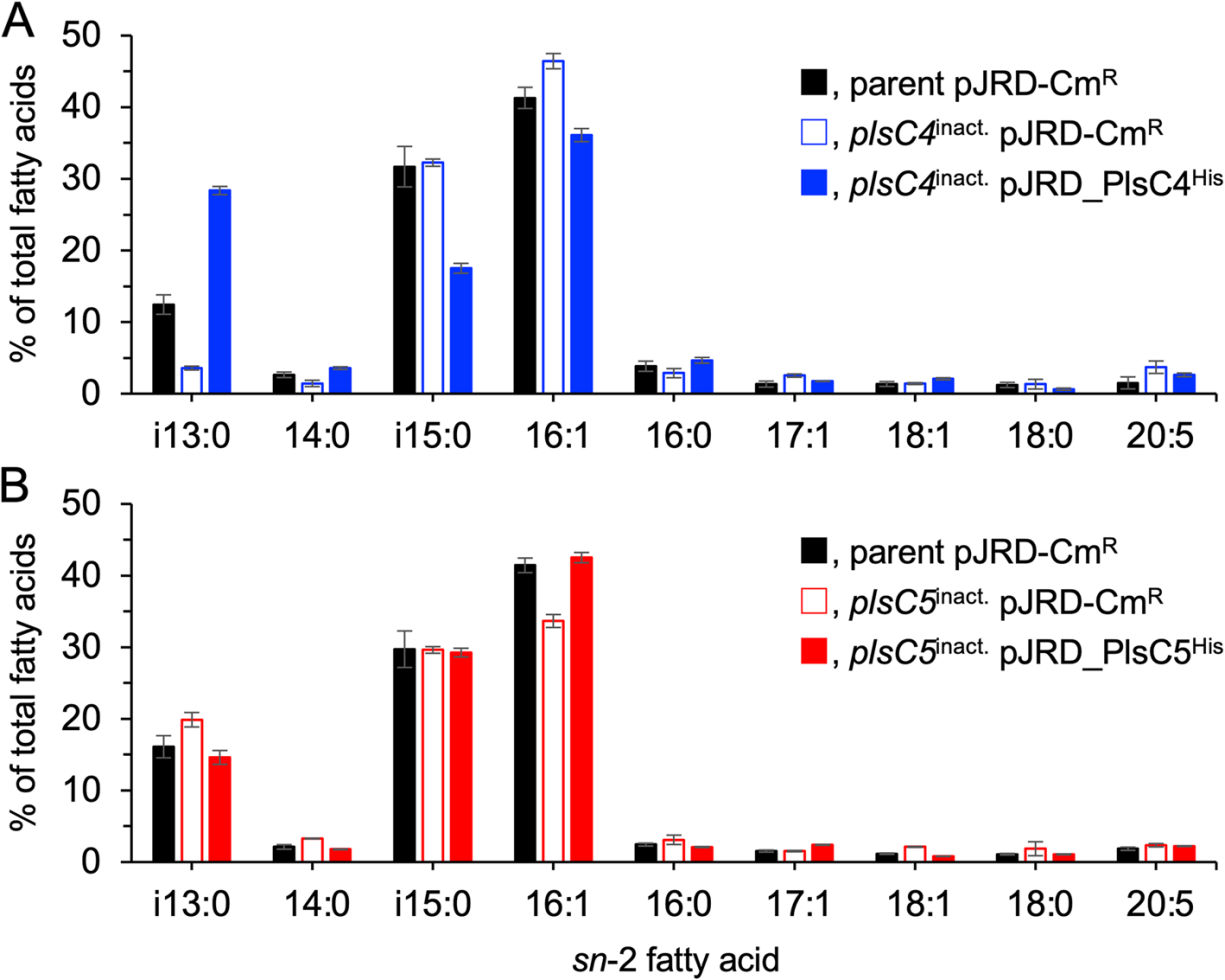
GC-MS analysis of *sn*-2 fatty acids. **A** Relative abundances of each fatty acid at *sn*-2 position in the parent cells harboring pJRD-Cm^R^ (closed black bar) and *plsC4*^inact.^ cells harboring pJRD-Cm^R^ (open blue bar) and pJRD_PlsC4^His^ (closed blue bar). **B** Relative abundances of each fatty acid at the *sn*-2 position in the parent cells harboring pJRD-Cm^R^ (closed black bar) and *plsC5*^inact.^ cells harboring pJRD-Cm^R^ (open red bar) and pJRD_PlsC5^His^ (closed red bar). The data were obtained from three independent experiments

Generally, the stereochemical positions of the fatty acyl groups cannot be accurately determined from the MS/MS spectra of PLs. Therefore, to further verify the substrate preference of PlsC4 and PlsC5, PL extracts were treated with phospholipase A2 to specifically hydrolyze the *sn*-2 ester bond, and the released *sn*-2 fatty acids were methyl-esterified and subjected to GC-MS analysis. The results demonstrated that i13:0 and 14:0 levels were 3.6 and 1.4% of the total fatty acids in the *plsC4*^inact.^ mutant, and were 3.5- and 1.8-fold lower than the parental levels, respectively (Fig. 4A). The amount of i15:0 fatty acid remained unchanged upon inactivation, while the amounts of 16:1, 17:1, and 20:5 fatty acids increased by 1.1-, 1.9-, and 2.5-folds, respectively. The complementation significantly enhanced the i13:0 and 14:0 levels with a concomitant decrease in i15:0 and 16:1. Thus, i13:0 was the preferred substrate for PlsC4.

*plsC5* inactivation caused only a modest change in *sn*-2 fatty acid composition. The *plsC5*^inact.^ mutant demonstrated a lower level of 16:1 fatty acid than the parent cells by approximately 20% (Fig. 4B). In contrast, the levels of fatty acids i13:0, 14:0, and 18:1 increased 1.2-, 1.6-, and 1.8-folds, respectively, in the mutant. Gene complementation reverted these alterations to levels similar to the parental level. These data indicated that 16:1 was utilized by PlsC5 as the acyl donor substrate.

### Phylogenetic analysis of PlsC4 and PlsC5

The function of the putative acyltransferase YihG in *E. coli* remained unknown for a long time. However, we recently found it to be a putative ortholog of PlsC4 (39% identical) and demonstrated it as a novel LPAAT with a substrate specificity different from that of PlsC from *E. coli* [12]. As a BLAST search indicated that other bacteria such as *Pseudomonas* and *Vibrio* also have putative PlsC4/YihG ortholog(s), we expanded the scope of an ortholog search using the KEGG Ortholog Cluster database. PlsC4 and PlsC5 orthologs from *Shewanella* species and *E. coli* YihG were grouped into the cluster OC.1452651, which includes 979 acyltransferase homologs primarily from γ-proteobacteria and several species of β- and δ-proteobacteria. This database indicates that many, but not all, γ-proteobacteria possess one or more PlsC4/PlsC5 homologs. For instance, *E. coli*, *Salmonella enterica*, and *Vibrio cholerae* have one homolog frequently annotated as YihG, whereas *Pseudomonas fluorescens* and *Aeromonas hydrophila* have two homologs (Table 1). In addition, similar to *S. livingstonensis* Ac10 (Fig. 1B), a pair of *plsC5-plsC4* genes is conserved in some marine bacteria, such as *Pseudoalteromonas atlantica*, *Moritella viscosa*, *Glaciecola nitratireducens*, and *Ferrimonas balearica* and tandemly aligned in their genomes, as indicated in Table 1, where the 3′-end of *plsC5* and 5′-end of *plsC4* were spaced by a few nucleotides (in *P. atlantica*), were located adjacent to each other with no spacer (in *F. balearica*), or overlapped with each other (in *M. viscosa* and *G. nitratireducens*).

**Table 1:** LPAAT homologs used in the phylogenetic analysis

Bacterial species	OC.1452651		PlsC
	PlsC4/PlsC5	other lineages	
Alteromonadales			
*Shewanella livingstonensis* Ac10	Sl_PlsC4 (BBD74888) (BDP99515)		Sl_PlsC1 (BBC27597)
*Colwellia psychrerythraea* 34H	(AAZ28101)		
*Ferrimonas balearica* DSM 9799	(ADN77849) Fbal_3654 (ADN77850)		
*Moritella viscosa* MVIS1	(CED60802) (CED62263) MVIS_4387 (CED62264)		
*Pseudoalteromonas atlantica* T6c	(ABG42554) Patl_4056 (ABG42555)		
*Pseudoalteromonas haloplanktis* TAC125	(CAI87881)		
*Marinobacter hydrocarbonoclasticus* VT8		Maqu_2692 (ABM19767) Maqu_3794 (ABM20863)	
*Glaciecola nitratireducens* FR1604	GNIT_0806 (AEP28950) (AEP28951)		
*Idiomarina loihiensis* L2TR	IL2569 (AAV83401)		
Vibrionales			
*Vibrio cholerae* O1 El Tor N16961	VC0033 (AAF93211)		
*Allivibrio fischeri* ES114	VF_2554 (AAW87049)		
*Vibrio parahaemolyticus* RIMD 2210633	VP3055 (BAC61318)		
Aeromonadales			
*Aeromonas hydrophila* ATCC 7966	(ABK38579) AHA_4209 (ABK39545)		
*Tolumonas auensis* DSM 9187	Tola_3051 (ACQ94640)		
Oceanospirillales			
*Thalassolituus oleivorans* MIL-1		TOL_1054 (CCU71488)	
*Halomonas elongata* DSM 2581		HELO_2534 (CBV42418)	
Enterobacteriales			
*Escherichia coli* K-12 MG1655	Ec_YihG (AAC76860)		Ec_PlsC (AAC76054)
*Klebsiella pneumoniae* MGH78578	KPN_04171 (ABR79550)		
*Salmonella enterica* Typhimurium LT2	STM3998 (AAL22837)		
Pseudomonadales			
*Pseudomonas fluorescens* F113		PSF113_4891 (AEV64880) PSF113_5421 (AEV65393)	Pf_HdtS (AEV60042) Pf_PatB (AEV64097)
*Acinetobacter baumannii* ATCC 17978		A1S_0883 (ABO11315)	
*Moraxella catarrhalis* BBH18		MCR_1596 (ADG61853)	
*Psychrobacter arcticus* 273–4		Psyc_1105 (AAZ18956)	
Cellvibrionales			
*Cellvibrio japonicus* Ueda107		CJA_1412 (ACE84611)	
Legionellales			
*Legionella pneumophila* Philadelphia 1		lpg0889 (AAU26976)	
*Coxiella burnetti* RSA 493		CBU_0027 (AAO89597) CBU_2073	
Thiotrichales			
*Francisella tularensis* SCHU S4		FTT_0180 (CAG44813)	

**Fig. 5:**
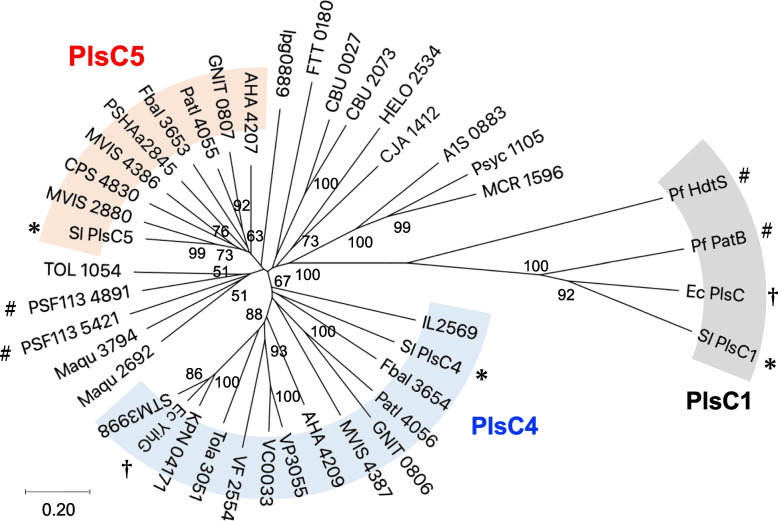
Phylogenetic analysis of PlsC4 and PlsC5. PlsC4 and PlsC5 homologs and well-characterized PlsCs from a broad range of γ-proteobacteria were grouped by a neighbor-joining method using MEGA X software. Symbols attached to proteins from *S. livingstonensis* Ac10 (asterisk), *E. coli* MG1655 (dagger), and *P. fluorescens* F113 (sharp). Bootstrap values greater than 50% are indicated for each node

The solid and wavy underlines indicate orthologs of PlsC4 and PlsC5, respectively, as deduced from the phylogenetic tree (Fig. 5). GenBank accession numbers are indicated in the parentheses

PlsC4 and PlsC5 are highly homologous to each other (39% identical), but distant from PlsC1 (~ 20% identical). We collected amino acid sequences of members of the PlsC4/PlsC5/YihG ortholog cluster and a few well-studied PlsCs [5, 14, 29] (as an outgroup) from a broad range of γ-proteobacteria (Table 1) and analyzed their phylogenetic relationships using Molecular Evolutionary Genetics Analysis (MEGA) X software [30]. The resulting phylogenetic tree demonstrated that PlsC4/YihG and PlsC5 homologs formed two distinct subtrees distant from a branch of principal LPAATs, including PlsC1 and *E. coli* PlsC (Fig. 5). PlsC4 orthologs from marine bacteria are phylogenetically similar to *E. coli* YihG and other homologs from Enterobacteriales, Vibrionales, and Aeromonadales. Therefore, it is now evident that YihG from *E. coli* and PlsC4 from *S. livingstonensis* Ac10 are orthologs that originate from the same evolutionary route. In contrast, the branch of PlsC5 comprised orthologs from Alteromonadales and *A. hydrophila*, indicating a narrower distribution among γ-proteobacteria than PlsC4/YihG. Among Alteromonadales marine bacteria, *Idiomarina loihiensis* has only a PlsC4 ortholog, whereas *Colwellia psychryrethraea* and *Pseudoalteromonas haloplanktis* have only a PlsC5 ortholog. Thus, PlsC4 and PlsC5 are not always paired, and their presence varies among bacterial species. Moreover, other lineages of the PlsC4/PlsC5/YihG homologs were found in the phylogenetic tree. For example, *P. fluorescens* F113 possesses two acyltransferases, PSF113_4891 and PSF113_5421, which do not belong to the subgroups PlsC4/YihG and PlsC5, in addition to two principal LPAATs designated as PatB and HdtS [5].

## Discussion

In this study, we investigated the enzymatic functions of PlsC4 and PlsC5 in *S. livingstonensis* Ac10 by generating *plsC4*^inact.^ and *plsC5*^inact.^ mutants and analyzing their PLs. Mutating a few codons of catalytic residues enabled us to evaluate the functions of PlsC4 and PlsC5 without inducing polar effects. ESI-MS and GC-MS analyses demonstrated that PlsC4 preferred a methyl-branched, medium-chain fatty acid, i13:0, but not i15:0, as the acyl donor substrate, whereas PlsC5 utilized a monounsaturated long-chain fatty acid, 16:1 (Figs. 3 and 4). The preference of PlsC4 towards i13:0 is unique because bacterial LPAATs that have been characterized thus far prefer fatty acids with carbon chain lengths of C_14_ or more [4–6, 14, 25]. The substrate selectivities of PlsC4 and PlsC5 differ from each other despite their high amino acid sequence identity, and also differ from that of PlsC1, which prefers polyunsaturated fatty acids [13, 14]. The ability to introduce a specific fatty acid into PLs is a key characteristic of LPAATs; however, the mechanism for substrate selection has not been clearly demonstrated. We expect that a comparative analysis of these enzymes will provide clues about the molecular basis underlying the recognition of different types of fatty acids by LPAATs. In contrast to the different acyl donor preferences, PlsC4 and PlsC5 likely shared LPA with i15:0 at the *sn*-1 position as the acyl acceptor substrate, given that 28:0-PE/PG and 31:1-PE/PG, which have i15:0 at the *sn*-1 position (Fig. S2A and S3A), were significantly reduced by the inactivation of *plsC4* and *plsC5* (Fig. 3).

This conclusion is not in line with our previous finding that PlsC4 uses i15:0 as the donor substrate [18]. In a previous study, levels of PE/PG with two i15:0 acyl groups and i15:0 level at the *sn*-2 position were considerably reduced in the Δ*plsC4* mutant but were not restored by complementation. We then assumed that PlsC4 prefers i15:0; however, in the complemented cells, owing to the overexpression of PlsC4, incorporation of i13:0 into PLs proceeded preferentially over its elongation to i15:0 via a fatty acid synthesis pathway, thus leading to a decrease in i15:0 levels. However, the results of the present study (Figs. 3 and 4) that exclude a polar effect are more reliable, and we now speculate that i15:0 was not the preferred substrate of PlsC4 and the reduced i15:0 level in the Δ*plsC4* mutant could be the consequence of a polar effect. It is also noteworthy that PlsC5 has an acyl acceptor substrate preference, especially towards i15:0-LPA: although the levels of 31:1-PE and 31:1-PG, which have i15:0 and 16:1 at the *sn*-1 and *sn*-2 positions, respectively, were reduced by 2-fold, those of 32:2-PE and 32:2-PG, which contain two 16:1 acyl groups, were not altered in *plsC5*^inact.^ mutants (Fig. 3B and S3). This property is supposed to facilitate a less significant reduction in the 16:1 level at the *sn*-2 position (Fig. 4B) than in the 31:1-PE and 31:1-PG levels (Fig. 3B). An in vitro characterization should be performed to determine the definite substrate selectivity of PlsC4 and PlsC5. Although purification of LPAATs is generally difficult because membrane solubilization using a detergent often compromises their enzymatic activity, we plan to investigate a method to purify these enzymes with various detergents, including the one we used to purify active PlsC1 [14].

Through phylogenetic analysis, we found that the PlsC4/YihG and PlsC5 subfamilies are distributed among γ-proteobacteria, and there are still other lineages of the homologs (Fig. 5, Table 1). These acyltransferases have been overlooked in previous studies because PlsC in *E. coli* and PatB/HdtS in *P. fluorescens* are indispensable, and members of the PlsC subfamily have been regarded as essential LPAATs in bacteria [5, 11]. Thus, the production of PLs with different fatty acid compositions by LPAATs and their biological significance have been discussed based on the multiplicity of principal LPAATs alone [3–6]. However, given that PlsC4, PlsC5 (Fig. 3), and *E. coli* YihG contribute to PL formation, the significance of their homologs in membrane lipid synthesis should also be considered. Some bacteria possess a pair of PlsC4 and PlsC5 orthologs, whereas others have either of the two orthologs. The paired genes are closely adjacent to or overlapping with each other in various genomes, suggesting that *plsC4* and *plsC5* are co-expressed and may be involved in the same physiological event that occurs in the lipid membrane. Additionally, these genes are frequently adjacent to genes that putatively encode a magnesium chelatase (in *Shewanella*, *Moritella*, *Ferrimonas*, *Aeromonas*, and *Vibrio*) or a mechanosensitive channel (in *Pseudoalteromonas*, *Colwellia*, *Glaciecola*, and *Idiomarina*). Although the relevance of the former protein to the lipid membrane is unknown, the gating activity of the latter is affected by changes in membrane thickness and curvature [31, 32]. Fatty acyl chains that have a shorter carbon chain length, a bulky methyl branch at the ω-terminal, and a kink of *cis*-C=C bond disorder the lipid membrane and enhance its deformability to varying degrees [7, 8]. Therefore, the introduction of i13:0 and 16:1 into membrane PLs by PlsC4 and PlsC5, respectively, should have a distinct impact on channel activity. Thus, PlsC4/PlsC5 may cooperatively regulate PL composition to control the membrane structure and function of membrane proteins.

The *plsC4*^inact.^ and *plsC5*^inact.^ mutants, which display no appreciable polar effects, would be useful for phenotypic analysis to dissect the physiological functions of PlsC4/PlsC5 and their PL products. The *plsC4*^inact.^ mutant was more sensitive to cold stress than the parent cells (Fig. 2B), suggesting that the i13:0-containing PLs produced by PlsC4 contribute to the survival of *S. livingstonensis* Ac10 in cold habitats. In contrast, both *plsC4*^inact.^ and *plsC5*^inact.^ mutants exhibited no change in cell motility (Fig. S1). This is different from the phenotype of the *E. coli yihG* mutant, which is more motile than the wild-type cell and displays elevated flagellar expression [12]. *E. coli* YihG differs from PlsC4/PlsC5 in its substrate selectivity towards 18:1 [12]. Taken together, these results suggest that members of the PlsC4/PlsC5/YihG subfamily may have evolved to acquire LPAAT activity with distinct properties and play varying physiological roles in different bacteria.

## Conclusions

In this study, we demonstrated that PlsC4 and PlsC5 from *S. livingstonensis* Ac10 have different substrate specificities and produce PLs with the *sn*-2 position acylated with i13:0 and 16:1, respectively. Considering the different effects on low-temperature growth of *S. livingstonensis* Ac10, the physiological roles of PlsC4 and PlsC5 are also different. In addition, we found that many γ-proteobacteria have LPAATs that belong to the PlsC4 and PlsC5 subfamilies and some close lineages, in addition to principal LPAATs, suggesting that the division of the role of LPAAT paralogs is more common and complicated than previously considered. The fatty acid composition of PLs affects the physicochemical properties of the PL membrane and the folding and function of PL-interacting membrane proteins. Therefore, although enzymes belonging to PlsC4/PlsC5-related subfamilies have been overlooked, both this novel clade of LPAATs and principal ones should be considered to decipher the PL metabolism and membrane physiology of bacteria.

## Methods

### Bacterial strains and culture conditions

The *pyrF*-deficient mutant (Δ*pyrF*) of *S. livingstonensis* Ac10 [26], which is a uracil (Ura) auxotroph and resistant to 5-fluoroorotic acid (5-FOA) toxicity, was used as a parent strain for mutagenesis. The *E. coli* DH5α strain was used for plasmid construction and the S17–1/λ*pir* strain was used as a donor cell for plasmid conjugal transfer. The bacteria were cultured in LB media (tryptone 10 g/L, yeast extract 5 g/L, and sodium chloride 10 g/L) that was, when indicated, supplemented with Ura, rifampicin (Rf), kanamycin (Km), chloramphenicol (Cm), and 5-FOA at a final concentration of 40 μg/mL, 50 μg/mL, 30 μg/mL, 30 μg/mL, and 0.1%, respectively. Bacterial growth in the LB medium was monitored by measuring the optical density at 600 nm (OD_600_). For the cell motility assay, cultured cells were spotted on a soft 0.2% agar plate of Marine Broth 2216 (BD Difco, Franklin Lakes, NJ, USA) and incubated for 20 days at 4 °C, and formation of halos attributed to bacterial cell swimming was observed.

### Generation of *plsC4*^inact.^ and *plsC5*^inact.^ mutants

Mutations to inactivate *plsC4* and *plsC5* were introduced into the genomic DNA via a two-step single-crossover recombination method [26], which is described as follows: The left and right arms (approximately 500 bp each) corresponding to upstream and downstream flanking regions of the codons that encode an acyltransferase motif I (His95-Asp100 of PlsC4 and His92-Asp97 of PlsC5) were amplified by PCR using the genomic DNA of *S. livingstonensis* Ac10 as the template and the following primer pairs (Fig. S4 and Table 2): primers 1 and 2 for the left arm of *plsC4*, primers 3 and 4 for the right arm of *plsC4*, primers 5 and 6 for the left arm of *plsC5*, and primers 7 and 8 for the right arm of *plsC5*. The plasmid pKKP [26] was amplified by PCR using the primers 9 and 10 (Table 2), in which primer extension proceeds outward from the SmaI site and the whole vector is amplified to generate linearized pKKP (Fig. S4). The resulting left and right arm fragments and linearized pKKP, which have 15-nucleotide overlapping sequences at their 5′- and 3′-ends, were fused using an In-Fusion cloning kit (TaKaRa Bio, Kusatsu, Shiga, Japan) following the manufacturer’s instructions to obtain plasmids pKKP-*plsC4*^inact.^ and pKKP-*plsC5*^inact.^. Through a plasmid conjugal transfer using *E. coli* S17–1/λ*pir* as the donor cell, Δ*pyrF* cells of *S. livingstonensis* Ac10 were transformed with the plasmids and selected on an LB agar plate supplemented with Rf, Km, and Ura for the 1st recombination. After a few rounds of single colony isolation, a Km-resistant clone was screened on an LB agar plate supplemented with Rf, 5-FOA, and Ura for the 2nd recombination. The resulting 5-FOA-insensitive *plsC4*^inact.^ and *plsC5*^inact.^ mutants were tested by colony PCR using primer pairs 1 and 12 (for *plsC4*^inact.^) or 11 and 13 (for *plsC5*^inact.^) (Table 2), which specifically amplify the mutant genome. For the *plsC5*^inact.^ mutant, additional screening was performed in which the colony PCR products obtained with primers 11 and 8 were digested with SalI. Since only the product from the wild-type has a SalI site, this method allows us to distinguish between the wild-type and mutant strains (see Fig. 2). To confirm successful mutation, the mutant genomic DNA was extracted, PCR-amplified using the primer pairs 1 and 14 (for *plsC4*^inact.^) or 11 and 15 (for *plsC5*^inact.^) (Table 2), and sequenced.

**Table 2:** Primers used in the present study

Primer		Sequence (5′ to 3′)
Inactivation of *plsC4* and *plsC5*		
1	*plsC4*_L-Fw	ACTAGTGGATCCCCCCTGATACCGTACCGACTTATTGG ^a^
2	*plsC4*_L-Rv	
3	*plsC4*_R-Fw	
4	*plsC4*_R-Rv	GAATTCCTGCAGCCCGCTTGAAAAGTAAACTTCTGAATCC ^a^
5	*plsC5*_L-Fw	ACTAGTGGATCCCCCGTTGTCAGTACGTAGTTTTCATC ^a^
6	*plsC5*_L-Rv	
7	*plsC5*_R-Fw	
8	*plsC5*_R-Rv	GAATTCCTGCAGCCCAAATTCGCGATCTTTCAGATAATCG ^a^
9	pKKP_inv_Fw	GGGCTGCAGGAATTCGATATC ^a^
10	pKKP_inv_Rv	GGGGGATCCACTAGTTCTAGAG ^a^
11	*plsC5/4*_check_Fw	CGATTACTCAGCCAGATCTGG
12	*plsC4*_check_Rv	AATGGCAAAACCTGATACTGC
13	*plsC5*_check_Rv	GCTACCCAGCTCTGTGC
14	*plsC4*_seq_Rv	CTGCGCTAGTACGATCCATAAAC
15	*plsC5*_seq_Rv	CAATATCTTTACCCTTCAACTTGGG
Complementation of *plsC5*^inact.^		
16	*plsC5*_Fw	
17	*plsC5*_Rv	GGACTAGTTCAGTGGTGGTG
18	LI3_Fw	CGACGCGTCGCGCATTCATAATCATT
19	LI3_Rv	

^a^ Solid underline represents the overlapping sequence for In-Fusion reaction

^b^ Boxed characters represent the nucleotides that code for the mutated catalytic residues

^c^ Wavy underline represents the overlapping sequence for overlap extension PCR

### Gene complementation

*plsC5* with a hexahistidine tag-coding sequence was amplified by PCR using the pET21a-derived PlsC5 expression plasmid [13] as the template and the primers 16 and 17 (Table 2), while a high-expression promoter LI3 [33] was amplified using the genomic DNA of *S. livingstonensis* Ac10 as the template and the primers 18 and 19 (Table 2). The resulting gene and promoter fragments had a 21-nucleotide overlapping sequence at their 5′- and 3′-end, respectively. To fuse them, overlap extension PCR was performed using the primers 17 and 18 under standard PCR conditions, in which an appropriate annealing temperature was set to allow the overlapping sequences to anneal to each other. The PCR product was inserted into the MluI/SpeI site of pJRD-Cm^R^ [18] to obtain pJRD_PlsC5^His^. Through plasmid conjugal transfer using *E. coli* S17–1/λ*pir* as the donor cell, a *plsC4*^inact.^ mutant was transformed with pJRD_PlsC4^His^ [18] and a *plsC5*^inact.^ mutant with pJRD_PlsC5^His^. Cm-resistant transformants were obtained by several rounds of single-colony isolation on an LB agar plate supplemented with Rf, Cm, and Ura.

### MS analysis of PL composition

Extraction and MS analysis of PLs were performed as described previously [18]. Briefly, the parent and mutant cells were cultivated in 10 mL LB medium, harvested at an OD_600_ of approximately 1.0, and then lyophilized. Dry cells were extracted with methanol/chloroform (2:1) using the Bligh-Dyer method [34]. The resulting lower phase containing PLs was collected, and an aliquot was dried under a stream of nitrogen gas and re-dissolved in 10 volumes of acetonitrile/methanol (2:1 v/v) containing 0.1% triethylamine. The PL solution was analyzed using a triple quadrupole tandem mass spectrometer API3000 (Sciex, Concord, Ontario, Canada) equipped with an ESI source.

### GC-MS analysis of *sn*-2 fatty acid composition

Cleavage of *sn*-2 fatty acids, methyl esterification, and GC-MS analysis were carried out as described elsewhere [18]. Briefly, the remaining PL extracts were dried, dissolved in 0.8 mL reaction buffer containing 0.1 M Tris-HCl (pH 9) and 5 mM CaCl_2_, and treated with 8 μL of phospholipase A2 solution (derived from the porcine pancreas; Merck, Darmstadt, Germany) at 37 °C for 4 h. The reaction was stopped by adding 3.5 mL of 2-propanol/*n*-hexane (4:1 v/v) containing 0.1% H_2_SO_4_, and the released *sn*-2 fatty acids were extracted using 3.5 mL of *n*-hexane/water (3:2 v/v). The hexane layer was collected and dried, and the residual fatty acids were methyl-esterified in 10% (w/v) HCl in methanol (0.5 mL). The resulting fatty acid methyl esters were extracted with 0.25 mL of dichloromethane and 1 mL of *n*-hexane and then analyzed using a gas chromatograph Clarus 680 interfaced with a Clarus SQ 8C mass spectrometer (Perkin Elmer, Waltham, MA, USA).

### Phylogenetic analysis of PlsC4/PlsC5 homologs

PlsC4 and PlsC5 orthologs were searched against the KEGG Ortholog Cluster database (https://www.genome.jp/tools/oc/) [35] using the amino acid sequence of PlsC4 as a query. The hit group OC.1452651 comprised 979 acyltransferase homologs as of September 2021, and included proteins corresponding to PlsC4 and PlsC5 from other *Shewanella* species. Amino acid sequences of several members of the cluster (listed in Table 1) were retrieved, and multiple sequence alignments were performed using the ClustalW program in MEGA X [30]. Molecular phylogenetic relationships of the members were then analyzed by the neighbor-joining method using MEGA X, in which a bootstrap value was calculated from 1000 trials.

## Supplementary Information

**Additional file 1.**

**Additional file 2.** .

**Additional file 3.**

**Additional file 4.**

**Additional file 5.**

**Additional file 6.**

## Data Availability

The datasets used and/or analyzed during the current study are available from the corresponding author on reasonable request.

## Abbreviations

5-FOA: 5-fluoroorotic acid
BCFA: Branched-chain fatty acid
EPA: Eicosapentaenoic acid
ESI: Electrospray ionization
GC: Gas chromatography
MEGA: Molecular evolutionary genetics analysis
MS: Mass spectrometry
LPA: Lysophosphatidic acid
LPAAT: Lysophosphatidic acid acyltransferase
PA: Phosphatidic acid
PE: Phosphatidylethanolamine
PG: Phosphatidylglycerol
PL: Phospholipid
