# Supplementary figures and images for: Division of the role and physiological impact of multiple lysophosphatidic acid acyltransferase paralogs
Source: BMC Microbiol. 2022 Oct 6;22:241. doi: 10.1186/s12866-022-02641-8 (PMC9541089; doi:10.1186/s12866-022-02641-8)

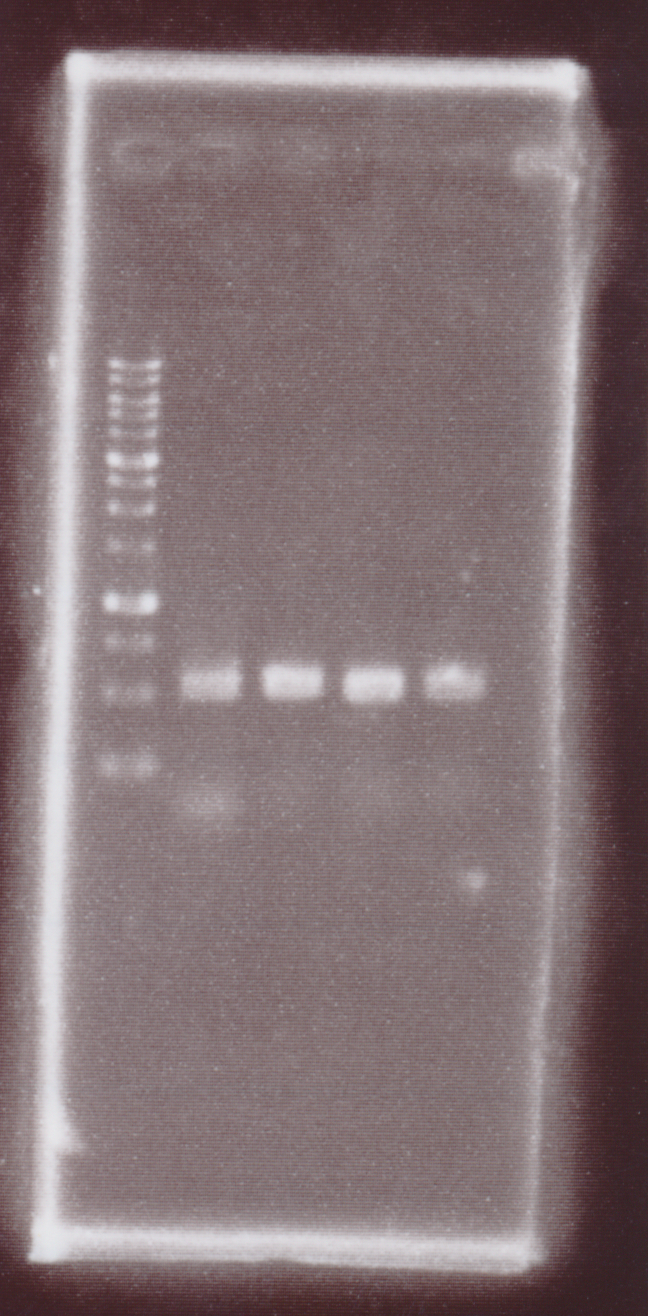

Supplement: Supplementary file 1 — Additional file 1. [file 12866_2022_2641_MOESM1_ESM.tiff]

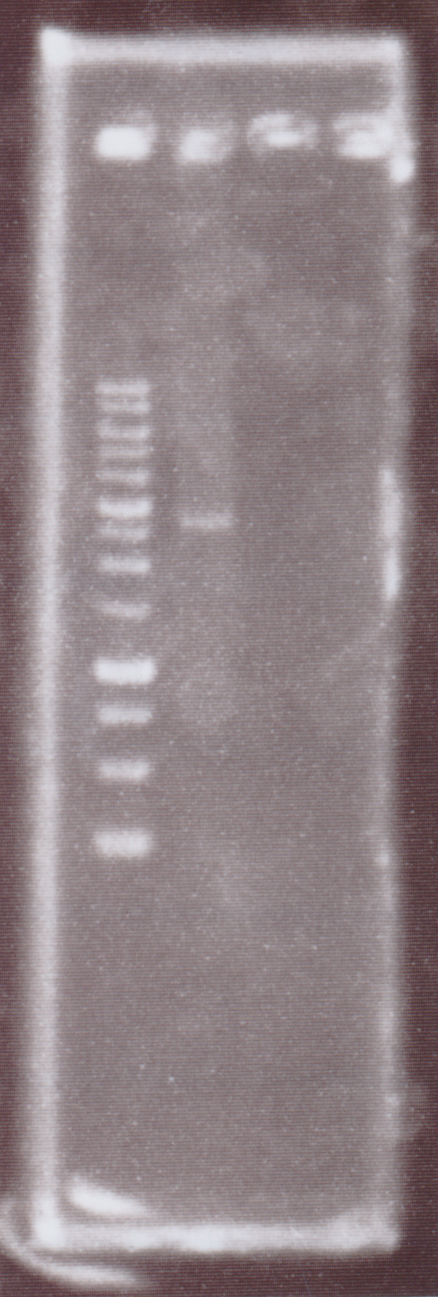

Supplement: Supplementary file 2 — Additional file 2. . [file 12866_2022_2641_MOESM2_ESM.tiff]

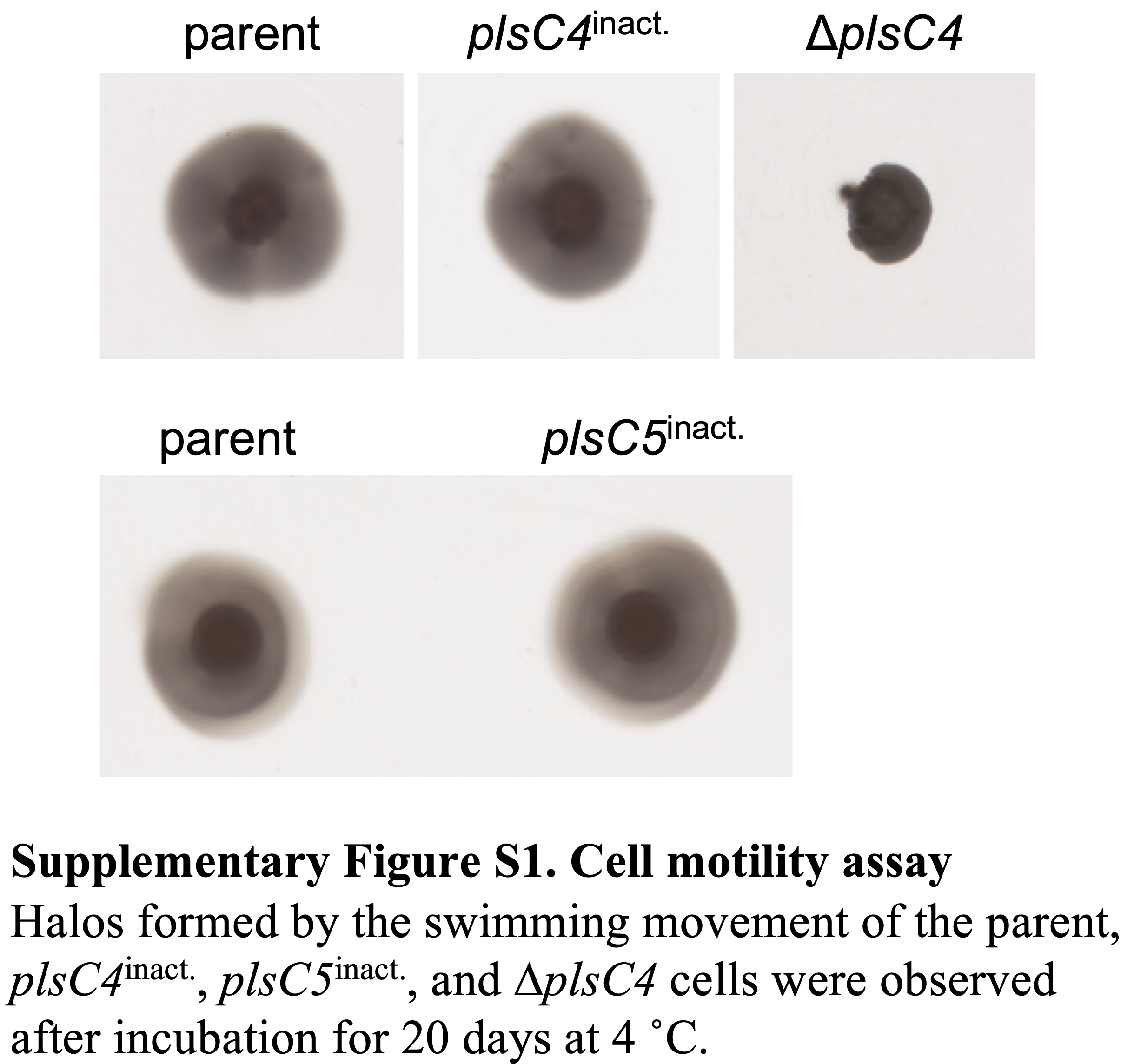

Supplement: Supplementary file 3 — Additional file 3. [file 12866_2022_2641_MOESM3_ESM.png]

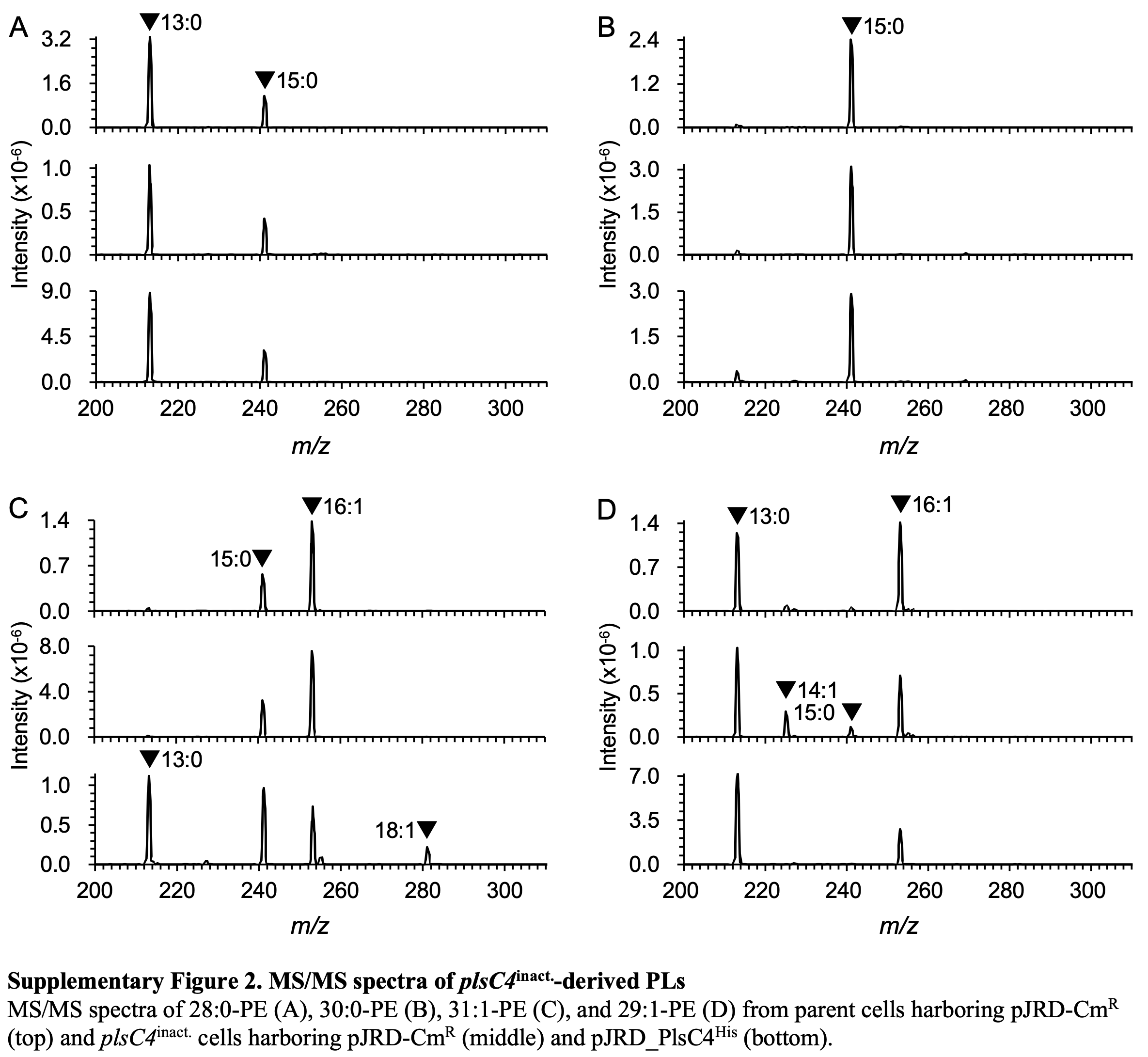

Supplement: Supplementary file 4 — Additional file 4. [file 12866_2022_2641_MOESM4_ESM.png]

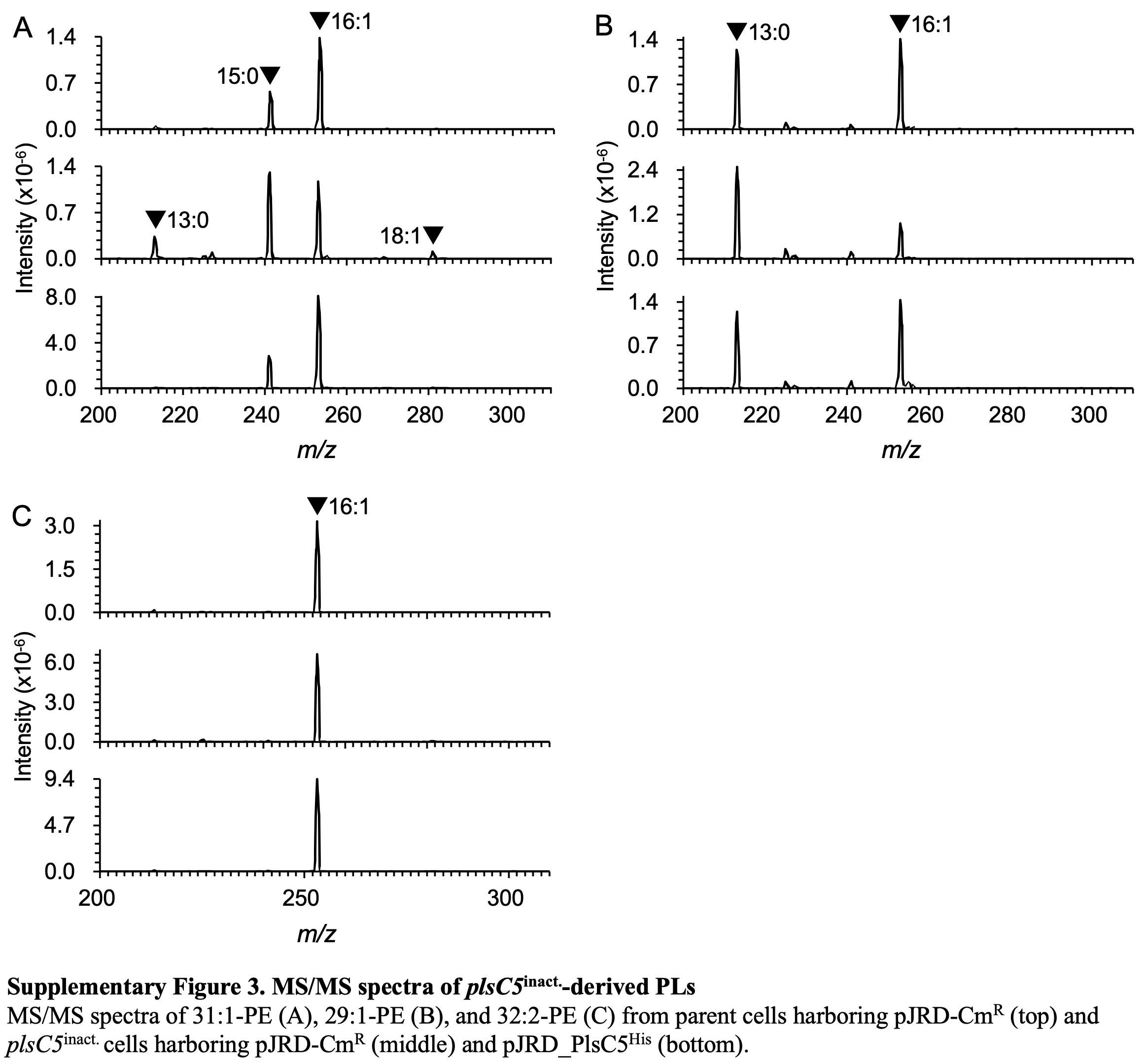

Supplement: Supplementary file 5 — Additional file 5. [file 12866_2022_2641_MOESM5_ESM.png]

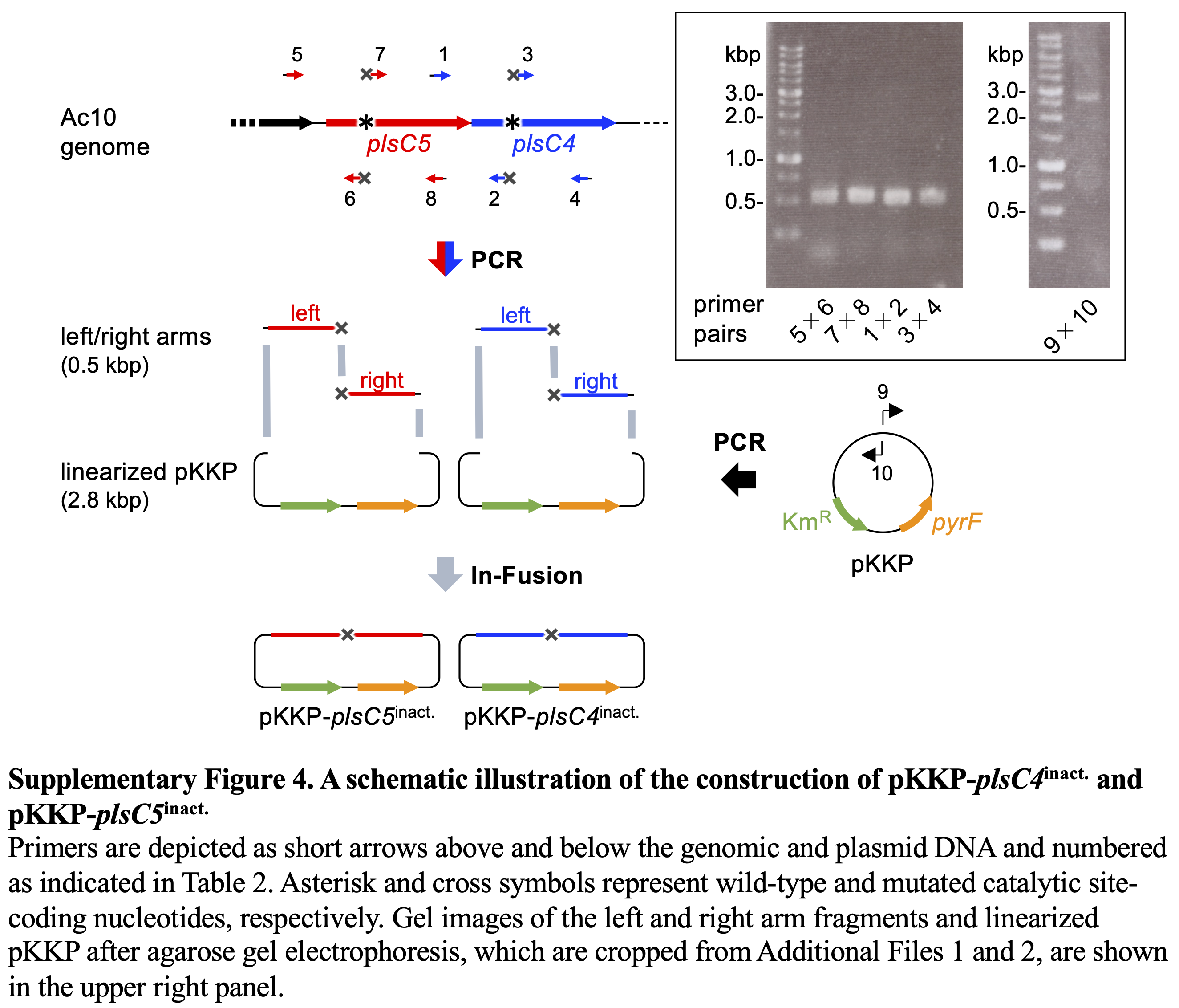

Supplement: Supplementary file 6 — Additional file 6. [file 12866_2022_2641_MOESM6_ESM.png]
